# Early Renal Perfusion Scintigraphy Is Associated with Mortality in Experimental Sepsis: A Multimodal Study Integrating Imaging, Survival, and Biomarker Analysis

**DOI:** 10.3390/diagnostics16162560

**Published:** 2026-08-14

**Authors:** Kubilay Kemertaş, Cengiz Dibekoğlu, Mert Zeytinoğlu, Hatice Aygun, Aylin Arslan, Serdar Savaş Gül, Oytun Erbas

**Affiliations:** 1 Department of General Surgery, Demiroğlu Bilim University, 34394 Istanbul, Turkey; drkemertas@gmail.com (K.K.); cdibekoglu@gmail.com (C.D.); 2 Department of Oral Surgery, Faculty of Dentistry, Ege University, 35040 İzmir, Turkey; mert.zeytinoglu@ege.edu.tr; 3 Neuroscience Laboratory, BAMER, Biruni University, 34010 Istanbul, Turkey; 4 Department of General Surgery, Prof. Dr. Cemil Taşçıoğlu City Hospital, 34384 Istanbul, Turkey; 5 Department of Nuclear Medicine, Faculty of Medicine, Lokman Hekim University, 06510 Ankara, Turkey; serdar.gul@lokmanhekim.edu.tr; 6 Faculty of Medicine, BAMER, Biruni University, 34010 Istanbul, Turkey; oytunerbas2012@gmail.com

**Keywords:** sepsis, cecal ligation and puncture, acute kidney injury, renal perfusion, scintigraphy, technetium-99m

## Abstract

Background/Objectives:This study aimed to evaluate whether early renal perfusion scintigraphy is associated with sepsis severity and short-term mortality in an experimental polymicrobial sepsis model. Methods: A total of 90 female Wistar albino rats were divided into Control, mild sepsis, and severe sepsis groups using a cecal ligation and puncture (CLP) model. Renal perfusion was evaluated 5 h after sepsis induction using technetium-99m-labeled erythrocyte scintigraphy, expressed as the kidney-to-aorta (R/A) activity ratio. Survival was monitored for 5 days. Histopathological and biochemical analyses were also performed. Results: The kidney-to-aorta (R/A) activity ratio decreased progressively with increasing sepsis severity (all *p* < 0.001). Kaplan–Meier analysis demonstrated significantly reduced survival in septic animals (log-rank *p* < 0.001). The R/A ratio showed high discrimination for 5-day mortality (AUC = 0.944, 95% CI 0.902–0.986; *p* < 0.001). Univariable Cox proportional hazards analysis demonstrated that a lower R/A ratio was significantly associated with an increased hazard of death (HR = 0.005, 95% CI 0.001–0.036; *p* < 0.001). Histopathological injury scores and biochemical markers (TNF-α, VEGF, STAT3, NGAL, BUN, creatinine, and MDA) increased significantly with sepsis severity. Conclusions: Early Tc-99m-labeled erythrocyte renal perfusion scintigraphy was associated with sepsis severity and short-term mortality in experimental polymicrobial sepsis. These findings support further investigation of this imaging approach as an experimental marker of sepsis severity and outcome; however, external validation is required before clinical translation.

## 1. Introduction

Sepsis is currently defined as life-threatening organ dysfunction caused by a dysregulated host response to infection, and it remains a major driver of preventable mortality despite advances in critical care [1,2,3]. Global estimates indicate an enormous and persistent burden, with tens of millions of cases and millions of deaths annually, highlighting the unmet need for earlier risk stratification and organ-protective interventions [4,5]. Modern sepsis guidelines therefore emphasize rapid recognition, prompt resuscitation, and timely escalation of care, yet the ability to anticipate which patients will deteriorate -particularly via kidney involvement- remains limited [6].

Sepsis-associated acute kidney injury (SA-AKI) is one of the most common and clinically important complications of sepsis, and it is closely associated with both early mortality and long-term renal outcomes [7,8]. Rather than being caused solely by reduced renal blood flow, SA-AKI reflects a complex interplay of microcirculatory disturbances, endothelial dysfunction, inflammation, and oxidative stress. Notably, these alterations may occur even when global renal perfusion appears relatively preserved, highlighting the importance of microvascular changes in disease progression [9,10].

Current diagnostic criteria for acute kidney injury rely mainly on serum creatinine and urine output [11]. However, serum creatinine is a relatively late marker and may be influenced by several factors during sepsis, limiting its usefulness in early detection [12,13]. Although biomarkers such as NGAL can detect tubular injury earlier, they are not entirely specific and may be affected by systemic inflammation [14,15]. In this context, functional imaging may provide additional insight by directly reflecting organ-level physiology. Renal scintigraphy, particularly when combined with vascular reference measurements such as the aorta, allows semi-quantitative assessment of renal perfusion [16]. Tc-99m-labeled erythrocytes, due to their intravascular distribution, may be especially useful for evaluating early perfusion changes.

The present study aimed to evaluate whether early erythrocyte-labeled renal perfusion scintigraphy could reliably reflect sepsis severity in a clinically relevant cecal ligation and puncture (CLP) model [17,18]. Different experimental sepsis severities were induced by varying the number of cecal punctures in the standardized CLP model. We specifically investigated whether the semi-quantitative kidney-to-aorta (R/A) activity ratio could distinguish between experimental severity groups and was associated with short-term mortality, while histopathological and biochemical findings were evaluated as complementary indicators of disease severity.

## 2. Materials and Methods

### 2.1. Animals

A total of 90 adult female Wistar albino rats (200–250 g) were used in this study. All procedures were approved by the Animal Experiments Local Ethics Committee of Gaziosmanpaşa University (Approval No: 51879863-106, Approval Date: 5 December 2013) and conducted in accordance with NIH guidelines. Animals were obtained from the university facility and acclimatized for one week under standard conditions (22 ± 2 °C, 50–60% humidity, 12-h light/dark cycle) with free access to food and water.

### 2.2. Experimental Design and Cecal Ligation and Puncture (CLP) Model

Rats were randomly assigned to three groups: Control (*n* = 30), mild sepsis (*n* = 30), and severe sepsis (*n* = 30). The control group underwent laparotomy only, without cecal ligation or puncture.

Sepsis was induced using the cecal ligation and puncture (CLP) model under sterile conditions. After a ~3 cm midline laparotomy, the cecum was exteriorized and ligated distal to the ileocecal valve with a 3-0 silk suture. To establish different severities, the cecum was punctured once with a 22-gauge needle in the mild group and twice in the severe group, followed by gentle extrusion of a small amount of fecal material to initiate polymicrobial infection. The cecum was then returned to the abdominal cavity, and the incision was closed in layers using 4-0 polyglactin sutures. Animals in the Control group underwent the same procedure without ligation or puncture. All CLP-treated animals received intraperitoneal 0.9% NaCl (30 mL/kg) within the first hour, followed by 10 mL/kg/day in two divided doses.

#### Experimental Groups

The animals were randomly allocated into the following experimental groups:

Group 1 (Control group, *n* = 30): Laparotomy only, without cecal ligation or puncture. The abdomen was closed under sterile conditions.

Group 2 (Mild sepsis, CLP, *n* = 30): Sepsis was induced by cecal ligation followed by a single puncture of the cecum.

Group 3 (Severe sepsis, CLP, *n* = 30): Sepsis was induced by cecal ligation followed by two punctures of the cecum to establish a more severe septic condition.

The experimental protocol lasted for 5 days, during which mortality was recorded. A total of 37 animals died during the follow-up period, including 13 from the mild sepsis group and 24 from the severe sepsis group.

No animals or data points were excluded on the basis of post hoc criteria. Survival and scintigraphic analyses included all 90 animals; terminal biochemical and histopathological analyses included only animals surviving to the scheduled day-5 sampling (Control, *n* = 30; Mild Sepsis, *n* = 17; Severe Sepsis, *n* = 6), as dictated by the experimental sampling schedule.

At the end of the experimental period, all surviving animals were deeply anesthetized with ketamine (75 mg/kg; Ketasol, Richterpharma AG, Wels, Austria) and xylazine (15 mg/kg; Rompun, Bayer, Leverkusen, Germany), followed by euthanasia via cervical dislocation. Blood samples were obtained by cardiac puncture for biochemical analyses, and kidney and liver tissues were harvested for subsequent histopathological evaluation.

### 2.3. Erythrocyte-Labeled Renal Perfusion Scintigraphy

Renal perfusion was evaluated in all animals 5 h after CLP induction using technetium-99m (Tc-99m)-labeled erythrocyte scintigraphy. For in vivo labeling, pyrophosphate (PYP) (TechneScan^®^ PYP, Mallinckrodt Medical, St. Louis, MO, USA, Catalog No: DRN 4342) was reconstituted with 5 mL of 0.9% saline at room temperature. Each rat received an intravenous injection of 0.1 mL PYP via the tail vein. After a 20-min incubation period to allow sufficient labeling of erythrocytes, 1 mCi Tc-99m was administered intravenously through the tail vein. Immediately following radiotracer administration, dynamic scintigraphic imaging was performed for 30 min using a gamma camera system (Mediso, AnyScan S, Budapest, Hungary).

Image analysis was performed semi-quantitatively by placing regions of interest (ROIs) over the kidneys and abdominal aorta on dynamic images. Time–activity curves were generated, and the measured imaging parameter was expressed as the kidney-to-aorta (R/A) activity ratio. This ratio was interpreted as a semi-quantitative index of relative renal blood-pool activity rather than a direct measurement of absolute renal blood flow. Bilateral kidney values were averaged for each animal. A blinded investigator performed all analyses.

### 2.4. Histological Staining

Kidney tissues were fixed in formalin, embedded in paraffin, sectioned at 4 µm, and stained with hematoxylin and eosin. Sections were examined under a light microscope (Olympus BX51, Olympus Corp., Tokyo, Japan), and images were captured using a digital camera system (Olympus C-5050, Tokyo, Japan).

Histological assessment was performed semi-quantitatively using an image analysis system (Image-Pro Express, version 1.4.5, Media Cybernetics, Rockville, MD,,USA). For each sample, ten randomly selected fields at ×20 magnification were independently evaluated by two blinded observers. Renal injury was assessed based on tubular necrosis, tubular dilatation, intraluminal debris, hemorrhage, and interstitial inflammation. Histological damage was graded according to the percentage of the affected area as follows: 0–5% (score 0), 6–20% (score 1), 21–40% (score 2), 41–60% (score 3), 61–80% (score 4), and 81–100% (score 5). Inter-observer agreement for H&E-based histopathological scoring was high (weighted Cohen’s κ = 0.86).

### 2.5. Biochemical Analysis

Blood samples collected into EDTA tubes were centrifuged at 3000 rpm for 10 min to obtain plasma, which was stored at −80 °C until analysis. Plasma levels of TNF-α, NGAL, and VEGF were measured using ELISA kits (Abcam, Cambridge, UK) according to the manufacturer’s instructions, and concentrations were calculated from standard curves.

Blood samples collected into serum-separator tubes were allowed to clot for 30 min at room temperature and then centrifuged at 3000 rpm for 10 min to obtain serum. Serum blood urea nitrogen (BUN) and creatinine concentrations were measured using an automated biochemical analyzer (Beckman Coulter LX-2000, Brea, CA, USA) according to the manufacturer’s instructions.

Kidney tissues were excised after euthanasia, rinsed with cold saline, and stored at −80 °C. Samples were homogenized in phosphate-buffered saline (PBS, pH 7.4) and centrifuged at 5000× *g* for 15 min at 4 °C. Supernatants were collected for analysis. Total protein levels were determined by the Bradford method, and STAT-3 levels were measured using ELISA. Results were normalized to protein content and expressed as pg/mg protein.

#### Assessment of Lipid Peroxidation

Lipid peroxidation was assessed by measuring plasma malondialdehyde (MDA) levels using the TBARS method. Plasma samples were mixed with thiobarbituric acid reagent, incubated at 100 °C for 60 min, then cooled and centrifuged. The absorbance of the supernatant was measured at 535 nm. MDA concentrations were calculated using a tetraethoxypropane standard curve and expressed as nmol/mL.

### 2.6. Statistical Analysis

Data were analyzed using SPSS (version 19) and GraphPad Prism (version 9). Normality was assessed with the Shapiro–Wilk test. Parametric data were expressed as mean ± SD and analyzed using one-way ANOVA followed by Tukey’s post hoc test. Non-parametric data were presented as median [interquartile range (IQR)] and analyzed using the Kruskal–Wallis test followed by the Mann–Whitney U test, as appropriate. Dynamic kidney-to-aorta (R/A) activity ratios were analyzed using two-way repeated-measures ANOVA followed by Tukey’s multiple-comparisons test. Body weight was analysed using a REML linear mixed-effects model with Holm-adjusted pairwise comparisons. Body weight differences between survivors and nonsurvivors were analyzed using the unpaired Mann–Whitney U test due to the non-normal distribution of the data. A two-tailed *p* value < 0.05 was considered statistically significant.

Receiver operating characteristic (ROC) analysis was performed in the entire experimental cohort (*n* = 90), including Control, mild-sepsis, and severe-sepsis animals, to evaluate the discriminatory performance of the kidney-to-aorta (R/A) activity ratio for 5-day mortality. The area under the ROC curve (AUC), 95% confidence interval (CI), and optimal cut-off value were calculated. The optimal cut-off value was determined using the maximum Youden index (sensitivity + specificity − 1) derived from the ROC curve coordinates. Univariable Cox proportional hazards regression analysis was performed to evaluate the association between the kidney-to-aorta (R/A) activity ratio and time to death. Survival distributions were estimated using the Kaplan–Meier method and compared using the log-rank test. A two-sided *p* value < 0.05 was considered statistically significant.

Sample size justification. The sample size was determined a priori based on our previous experience with the CLP model and published experimental studies using comparable polymicrobial sepsis protocols and biological endpoints, including inflammatory biomarkers, renal injury, and survival outcomes. Published CLP studies have commonly used group sizes of approximately 20–30 animals to evaluate these endpoints. Considering the expected biological variability of the CLP model and anticipated mortality during the 5-day follow-up period, 30 animals per group were considered appropriate to ensure sufficient statistical power for detecting biologically meaningful differences while maintaining compliance with ethical principles for animal research [17,18].

## 3. Results

### 3.1. Survival Analysis

**Figure 1:**
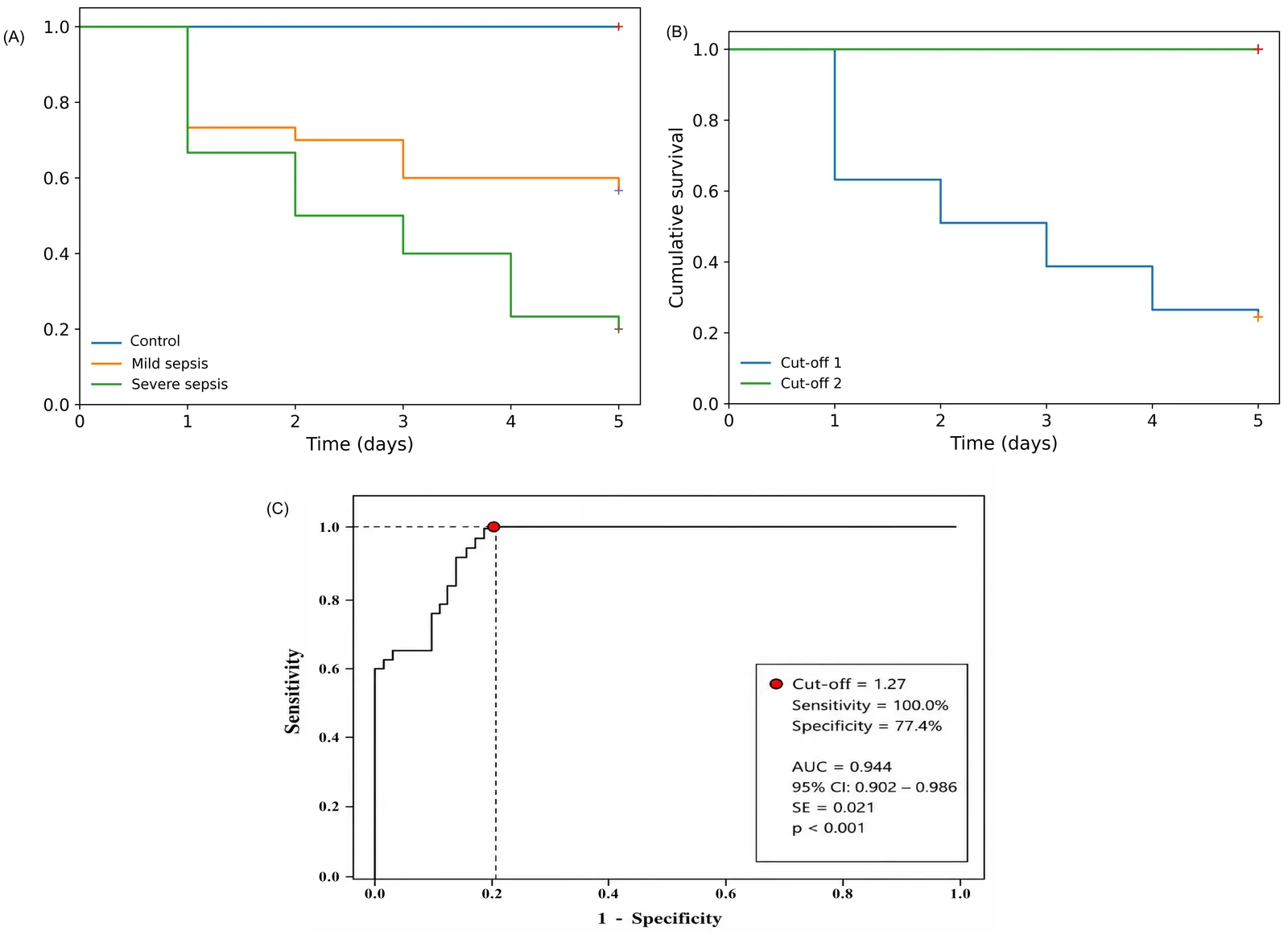
Kaplan–Meier survival curves and receiver operating characteristic (ROC) analysis of the kidney-to-aorta (R/A) activity ratio in relation to 5-day mortality in experimental polymicrobial sepsis. (**A**) Kaplan–Meier survival curves for the Control, mild sepsis, and severe sepsis groups during the 5-day follow-up. Survival differed significantly among groups (log-rank test, *p* < 0.001). (**B**) Kaplan–Meier survival curves stratified by the ROC-derived R/A ratio cutoff determined using the Youden index (R/A ≤ 1.274 vs. R/A > 1.274). Animals with lower R/A ratios had significantly lower survival than those with higher ratios (log-rank test, *p* < 0.001). (**C**) ROC curve of the R/A ratio for 5-day mortality. The AUC was 0.944 (95% CI: 0.902–0.986, *p* < 0.001), indicating high discrimination within the present cohort. The Youden-derived cutoff was 1.274, with 100.0% sensitivity and 77.4% specificity.

Kaplan–Meier survival analysis demonstrated significant differences in survival among the experimental groups (log-rank test, *p* < 0.001). Survival progressively decreased from the control group to the mild sepsis group and was lowest in the severe sepsis group. No mortality occurred in the control group during the 5-day follow-up period (Figure 1, Table 1).

Receiver operating characteristic (ROC) analysis demonstrated high discriminatory performance of the kidney-to-aorta (R/A) activity ratio for mortality (AUC = 0.944, 95% CI: 0.902–0.986, SE = 0.021, *p* < 0.001). Using the Youden index, the optimal cutoff value was identified as 1.274, yielding 100.0% sensitivity and 77.4% specificity. Lower kidney-to-aorta (R/A) activity ratios were associated with mortality (Figure 1).

Univariable Cox proportional hazards regression analysis demonstrated that the kidney-to-aorta (R/A) activity ratio was significantly associated with 5-day mortality (B = −5.384, SE = 1.052, Wald χ^2^ = 26.210, HR = 0.005, 95% CI: 0.001–0.036, *p* < 0.001). Higher kidney-to-aorta (R/A) activity ratios were associated with a lower hazard of death during the 5-day follow-up period (Figure 1 and Table 2).

### 3.2. Radiolabeled Red Blood Cell–Based Renal Perfusion Scintigraphy

**Figure 2:**
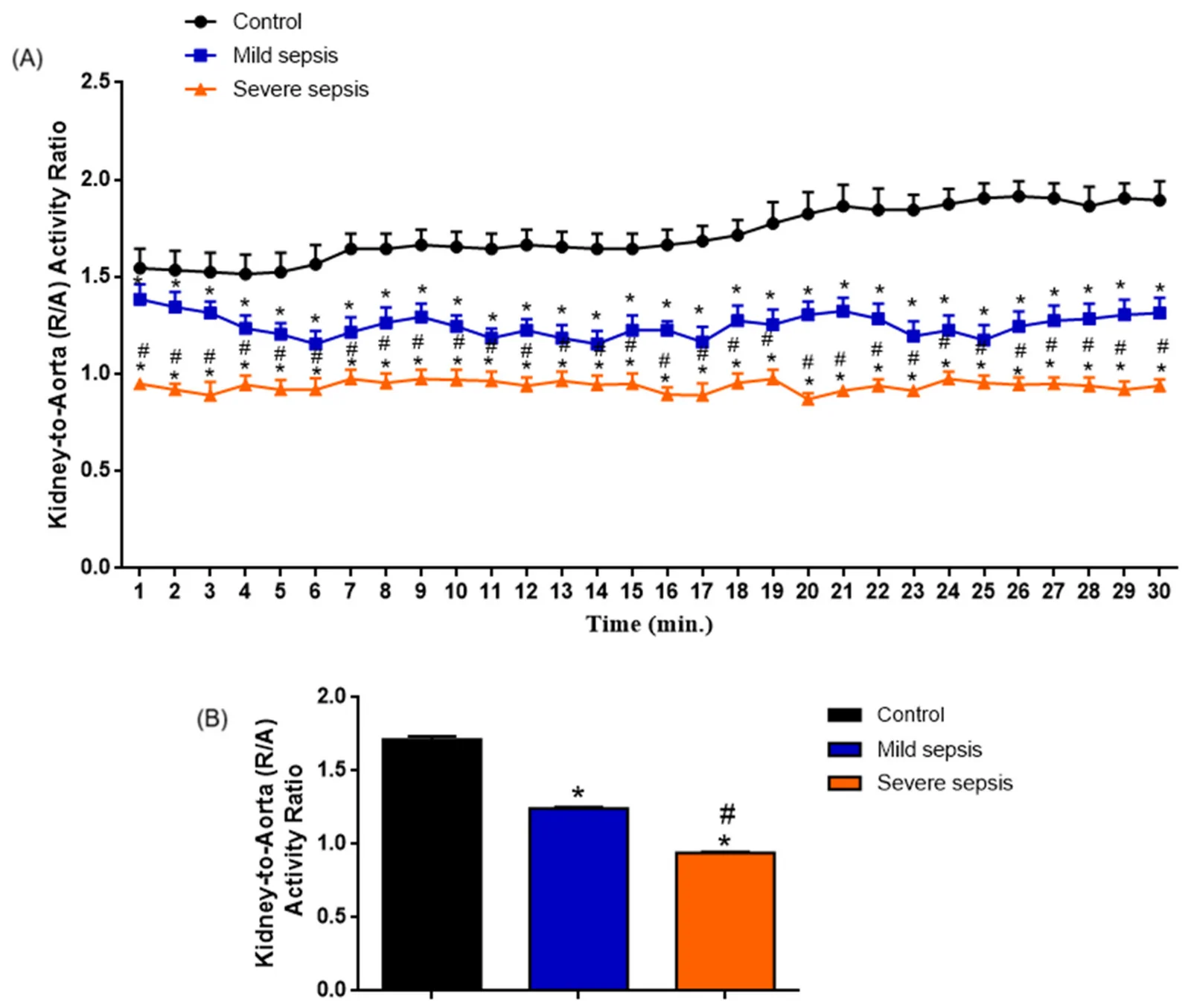
(**A**) Time-course of kidney-to-aorta (R/A) activity ratios over the 30-min acquisition period in the control, mild sepsis, and severe sepsis groups. (**B**) Mean kidney-to-aorta (R/A) activity ratios in the control, mild sepsis, and severe sepsis groups. Data are presented as mean ± SD. * *p* < 0.001 vs. control; # *p* < 0.001 vs. mild sepsis.

The kidney-to-aorta (R/A) activity ratio showed significant main effects of time (F(29,2523) = 65.95, *p* < 0.0001) and group (F(2,87) = 717.0, *p* < 0.0001), as well as a significant time × group interaction (F(58,2523) = 54.33, *p* < 0.0001). Post hoc Tukey’s multiple-comparisons test demonstrated that the control group had significantly higher kidney-to-aorta (R/A) activity ratios than both the mild and severe sepsis groups (all adjusted *p* < 0.0001). Furthermore, the mild sepsis group had significantly higher activity ratios than the severe sepsis group (all adjusted *p* < 0.0001). Overall, the kidney-to-aorta (R/A) activity ratio progressively decreased with increasing sepsis severity (Figure 2).

Kidney-to-aorta (R/A) activity ratios differed significantly among the groups (one-way ANOVA, F(2,87) = 717.0, *p* < 0.0001). Post hoc Tukey analysis revealed that all pairwise comparisons were statistically significant (*p* < 0.0001), with the control group showing the highest values, followed by the mild sepsis group, and the lowest values observed in the severe sepsis group (Figure 2B).

**Figure 3:**
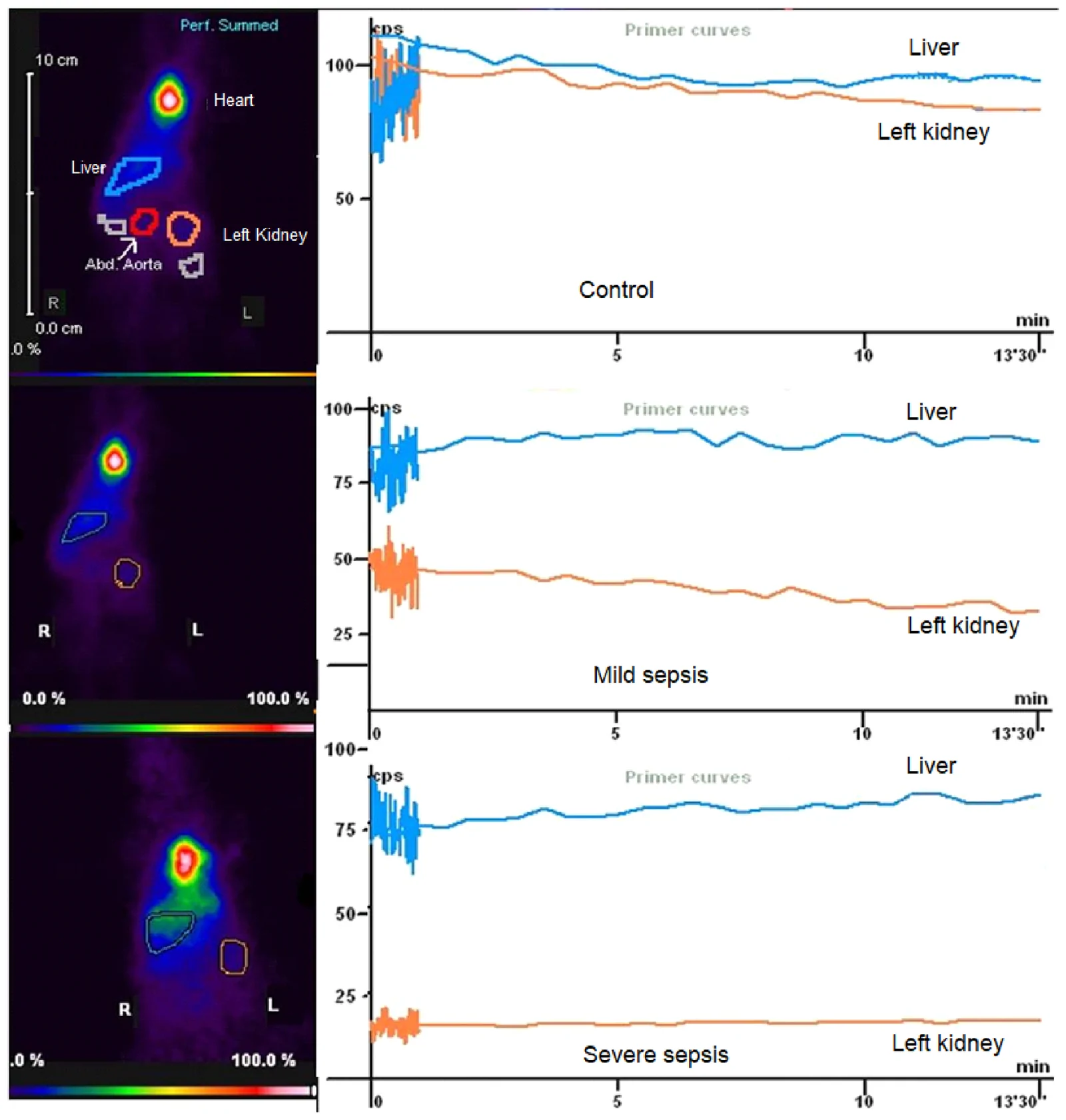
Representative renal blood-pool scintigraphic images and corresponding time–activity curves from the Control, Mild Sepsis, and Severe Sepsis groups. Scintigraphic images illustrate radiotracer distribution and the regions of interest (ROIs) used for quantitative analysis. The corresponding time–activity curves show representative liver and left kidney count profiles acquired during dynamic renal blood-pool scintigraphy.

In Figure 3, physiological radiotracer distribution with stable renal activity was observed in the control group. Renal activity was reduced in Mild Sepsis and markedly diminished with flattened time–activity curves in Severe Sepsis, consistent with reduced relative renal blood-pool activity. The kidney-to-aorta (R/A) activity ratio decreased in a severity-dependent manner, while hepatic uptake remained relatively preserved (Figure 3).

### 3.3. Histopathological Evaluation

**Figure 4:**
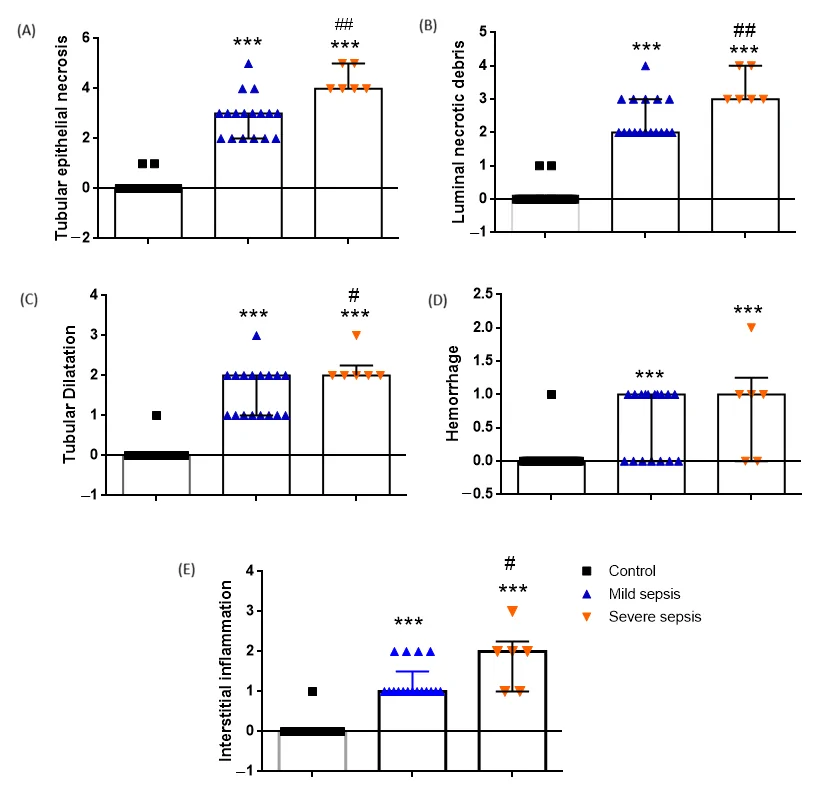
Histopathological injury scores in the Control, Mild Sepsis, and Severe Sepsis groups: (**A**) tubular epithelial necrosis, (**B**) luminal necrotic debris, (**C**) tubular dilatation, (**D**) hemorrhage, and (**E**) interstitial inflammation. Data are presented as medians with interquartile ranges (IQRs), with individual data points shown. *** *p* < 0.001 versus the Control group; # *p* < 0.05 and ## *p* < 0.01 versus the Mild Sepsis group.

**Figure 5:**
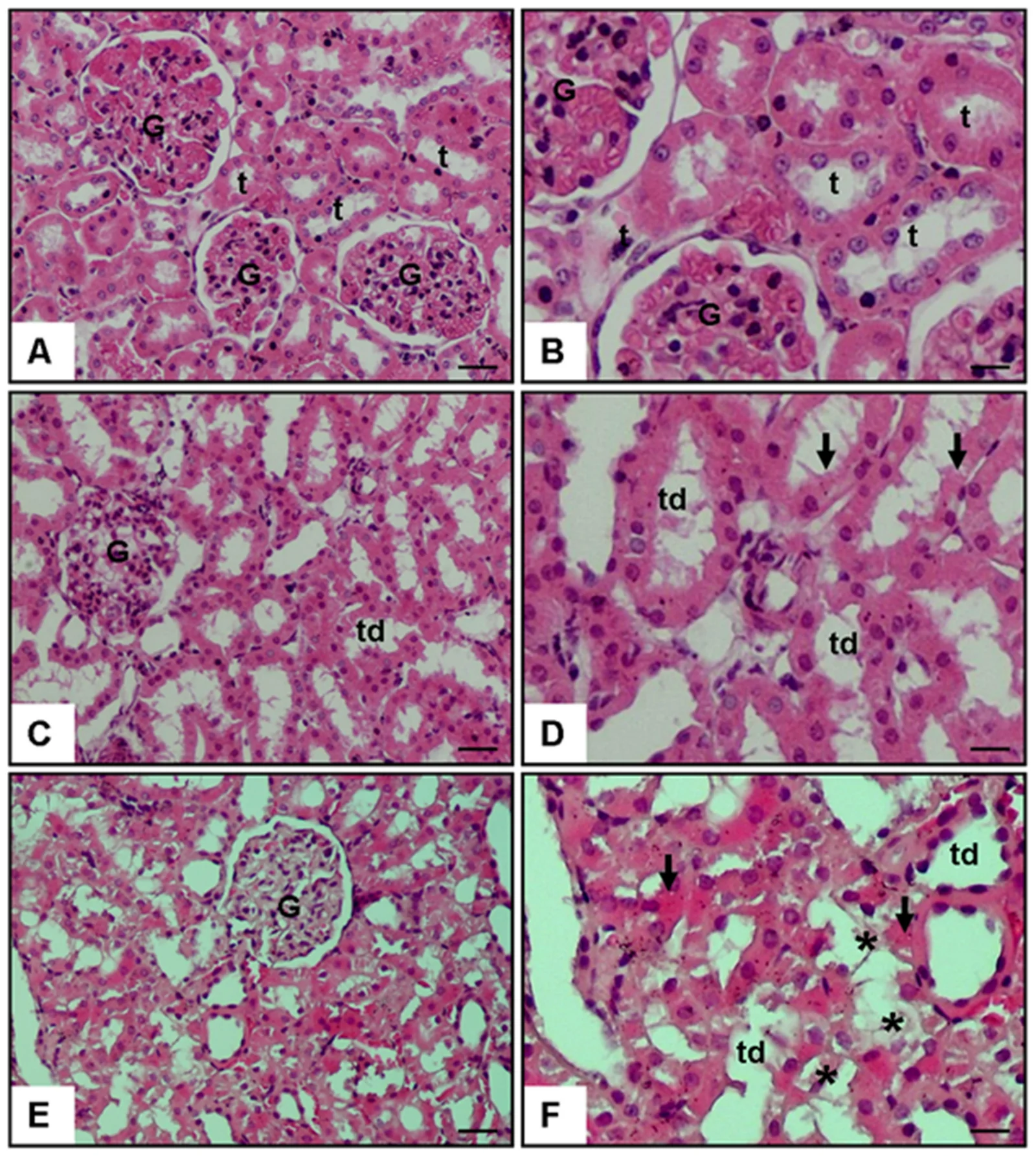
Kidney histopathology at ×20 and ×40 magnifications, hematoxylin and eosin (H&E) staining. (**A**,**B**): Control group demonstrating preserved renal architecture, including intact renal tubules (t) and normal glomeruli (G). (**C**,**D**): Mild sepsis group showing evident histopathological alterations characterized by tubular dilatation (td) and focal tubular epithelial necrosis (arrows). (**E**,**F**): Severe sepsis group exhibiting more pronounced renal injury, including marked tubular dilatation (td), widespread tubular epithelial necrosis (arrows), and accumulation of luminal necrotic debris (asterisks).

Histopathological analyses likewise included only animals surviving to the scheduled day-5 tissue collection (Control, *n* = 30; Mild Sepsis, *n* = 17; Severe Sepsis, *n* = 6). Kruskal–Wallis analysis showed significant differences among the Control, Mild Sepsis, and Severe Sepsis groups for tubular epithelial necrosis (χ^2^ = 47.267), luminal necrotic debris (χ^2^ = 47.162), tubular dilatation (χ^2^ = 46.667), hemorrhage (χ^2^ = 21.127), and interstitial inflammation (χ^2^ = 46.208) (all *p* < 0.001). Pairwise comparisons using the Mann–Whitney U test demonstrated that both the Mild Sepsis group and the Severe Sepsis group exhibited significantly higher histopathological injury scores compared to the Control group across all parameters (all *p* < 0.001). Furthermore, the Severe Sepsis group showed significantly greater tubular epithelial necrosis (*p* = 0.002), luminal necrotic debris (*p* = 0.006), tubular dilatation (*p* = 0.043), and interstitial inflammation (*p* = 0.044) compared to the Mild Sepsis group. In contrast, no significant difference was observed between the Mild Sepsis group and the Severe Sepsis group in terms of hemorrhage (*p* = 0.470) (Figure 4 and Figure 5).

### 3.4. Biochemical Analysis

**Figure 6:**
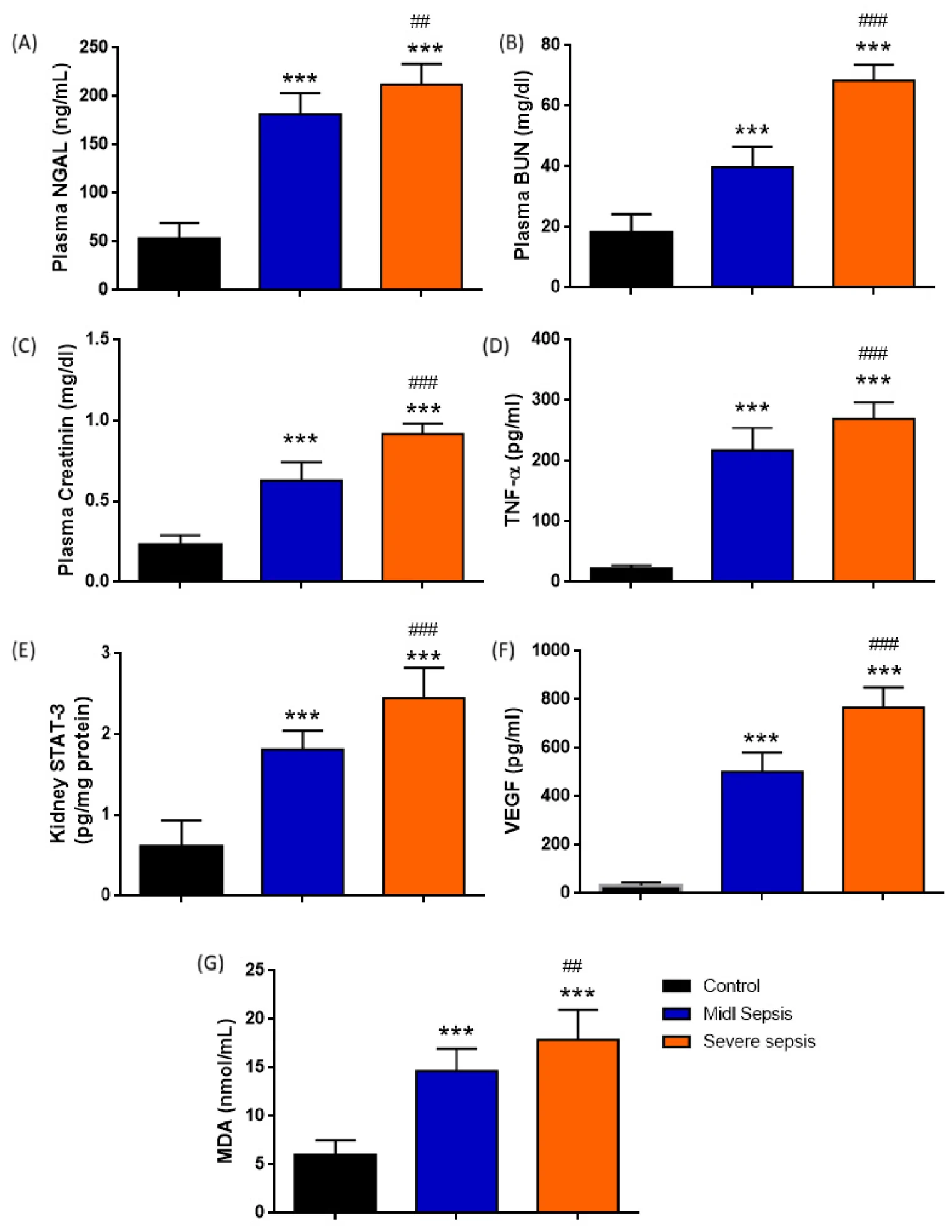
Biochemical parameters in the Control, Mild Sepsis, and Severe Sepsis groups: (**A**) plasma NGAL, (**B**) serum BUN, (**C**) serum creatinine, (**D**) plasma TNF-α, (**E**) kidney STAT3, (**F**) plasma VEGF, and (**G**) plasma MDA. Data are presented as mean ± SD (Control, *n* = 30; Mild Sepsis, *n* = 17; Severe Sepsis, *n* = 6). *** *p* < 0.001 versus the Control group; ## *p* < 0.01 and ### *p* < 0.001 versus the Mild Sepsis group.

Terminal biochemical analyses were restricted to animals surviving to day 5 (Control, *n* = 30; Mild Sepsis, *n* = 17; Severe Sepsis, *n* = 6). As shown in Figure 6, NGAL (F(2, 50) = 357.3), BUN (F(2, 50) = 184.5), and creatinine (F(2, 50) = 243.6) levels differed significantly among the Control, Mild Sepsis, and Severe Sepsis groups (all *p* < 0.0001). Both the Mild Sepsis and Severe Sepsis groups had higher levels than the Control group (all *p* < 0.0001). In addition, NGAL (*p* = 0.0032), BUN (*p* < 0.0001), and creatinine (*p* < 0.0001) levels were significantly higher in the Severe Sepsis group than in the Mild Sepsis group, indicating severity-dependent increases.

Significant differences were also observed among the Control, Mild Sepsis, and Severe Sepsis groups for TNF-α (F(2, 50) = 547.4), STAT3 (F(2, 50) = 143.2), and VEGF (F(2, 50) = 714.7) (all *p* < 0.0001). Both the Mild Sepsis and Severe Sepsis groups showed higher TNF-α, STAT3, and VEGF levels than the Control group (all *p* < 0.0001). Furthermore, TNF-α (*p* < 0.0001), STAT3 (*p* = 0.0001), and VEGF (*p* = 0.0001) levels were significantly higher in the Severe Sepsis group than in the Mild Sepsis group, indicating severity-dependent increases (Figure 6).

MDA levels differed significantly among the Control, Mild Sepsis, and Severe Sepsis groups (F(2, 50) = 149.5, *p* < 0.0001). Both the Mild Sepsis and Severe Sepsis groups showed higher MDA levels than the Control group (both *p* < 0.0001). In addition, MDA levels were significantly higher in the Severe Sepsis group than in the Mild Sepsis group (*p* = 0.0040), indicating a severity-dependent increase (Figure 6).

### 3.5. Body Weight

**Figure 7:**
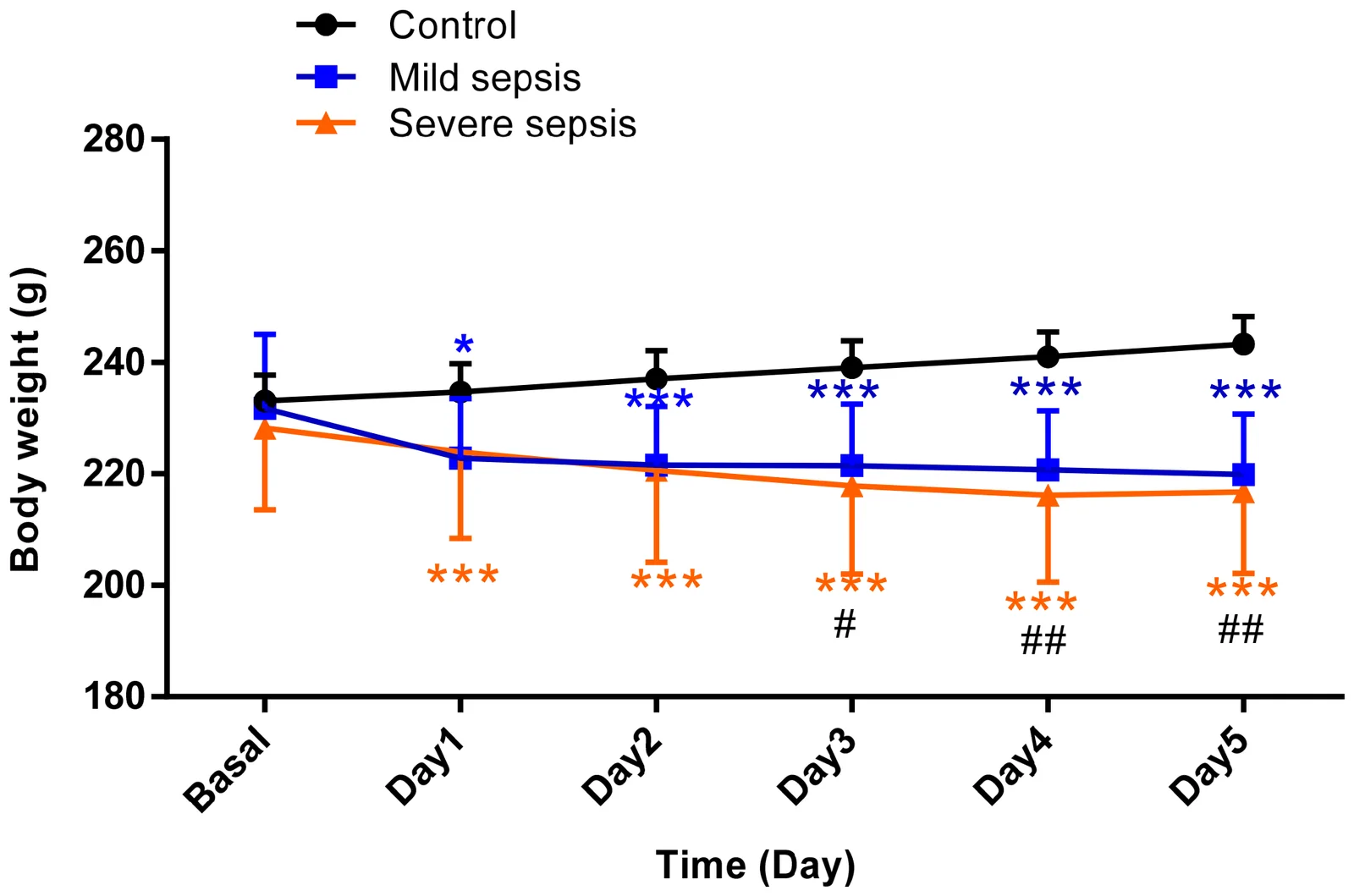
Body weight over time in the Control, Mild sepsis, and Severe sepsis groups. Data are presented as mean ± SD. Pairwise comparisons were adjusted using the Holm method. * *p* < 0.05, *** *p* < 0.001 versus the control group; # *p* < 0.05, ## *p* < 0.01, versus the Mild sepsis group. Baseline body weight did not differ significantly among the experimental groups (one-way ANOVA, *p* = 0.262).

Baseline body weight did not differ significantly among the experimental groups (one-way ANOVA, F(2,87) = 1.361, *p* = 0.262). The REML linear mixed-effects model demonstrated significant effects of group (Wald χ^2^ = 37.913, df = 2, *p* < 0.001), time (Wald χ^2^ = 179.655, df = 5, *p* < 0.001), and group × time interaction (Wald χ^2^ = 1270.376, df = 10, *p* < 0.001), indicating different body-weight trajectories among the experimental groups (Figure 7). Survivor-nonsurvivor comparisons showed a significant difference in the Mild Sepsis group (*p* = 0.031) but not in the Severe Sepsis group (*p* = 0.218); these findings should be interpreted cautiously because early mortality resulted in unequal follow-up durations.

## 4. Discussion

The principal finding of this study is that the kidney-to-aorta (R/A) activity ratio measured by Tc-99m-labeled erythrocyte scintigraphy 5 h after CLP decreased progressively across the control, mild sepsis, and severe sepsis groups and was associated with subsequent 5-day mortality. However, the ROC-derived cutoff requires external validation. Because the ROC analysis pooled control, mild sepsis, and severe sepsis animals and the cutoff was derived and evaluated within the same cohort, the high AUC and cutoff-based survival separation may partly reflect between-group case-mix and should be considered exploratory pending external validation. Moreover, because the Cox analysis was univariable, the R/A ratio should be interpreted as associated with mortality rather than as an independent predictor.

The distinction between the one- and two-puncture CLP protocols was supported by multimodal outcomes rather than puncture number alone. Rittirsch et al. [17] identified ligation length, needle diameter, puncture number, fecal extrusion, and perioperative management as key determinants of CLP severity. Dejager et al. [18] also emphasized the variability and translational limitations of the CLP model. In the present study, graded differences in survival, R/A ratio, histopathological injury, biochemical markers, renal function, and body-weight trajectories support the classification of the protocols as relatively mild and severe experimental sepsis phenotypes.

The timing of scintigraphy is biologically relevant because functional imaging may detect renal physiological changes before biochemical and structural injury becomes apparent. Experimental CLP studies have demonstrated early renal dysfunction and microvascular abnormalities [19,20], consistent with current concepts of SA-AKI involving inflammation, endothelial and microcirculatory dysfunction, and tubular metabolic adaptation despite relatively preserved global renal perfusion [8,21]. Perfusion-imaging studies similarly demonstrated renal microvascular or cortical hypoperfusion despite preserved systemic hemodynamics in experimental and clinical sepsis [22,23,24]. Thus, scintigraphy at 5 h may have captured an early functional phenotype; however, differences in imaging modalities and the absence of direct hemodynamic measurements preclude mechanistic interpretation or direct equivalence with these studies.

The progressive increases in TNF-α, STAT3, VEGF, and MDA further support the biological plausibility of the imaging findings. TNF-α is a key mediator of the early inflammatory response in sepsis and contributes to endothelial activation and organ injury [25,26,27]. Increased renal STAT3 expression is consistent with activation of cytokine-mediated inflammatory signaling [28,29]. Likewise, elevated VEGF and MDA levels support the presence of endothelial dysfunction and oxidative stress, two central mechanisms implicated in sepsis-associated acute kidney injury [30,31,32]. Collectively, these findings are consistent with current concepts of SA-AKI, in which inflammation, endothelial dysfunction, oxidative stress, and microcirculatory impairment interact to promote progressive renal injury [7,8].

The potential value of scintigraphy should be considered alongside established approaches to early SA-AKI detection. Serum creatinine is a delayed functional marker influenced by non-steady-state kinetics, baseline kidney function, creatinine generation, and volume of distribution [7,12,33]. NGAL may rise earlier and reflect tubular injury, but circulating NGAL is also released by activated neutrophils and may increase during sepsis independently of renal dysfunction, resulting in context-dependent thresholds and specificity [34,35,36,37]. In the present study, NGAL, BUN, and creatinine increased with experimental severity among animals available for terminal sampling, whereas the 5-h R/A ratio was associated with subsequent mortality. Together, these findings suggest that renal perfusion scintigraphy may complement established biomarkers. However, because biochemical measurements were obtained only at the terminal experimental time point in surviving animals, the present design cannot establish incremental prognostic value over NGAL, BUN, or creatinine owing to temporal mismatch and survivor-selection bias. Future studies should compare contemporaneous early imaging with serial biomarker measurements using prespecified multivariable models and independent validation.

The graded tubular injury observed on histopathology provides additional biological support for the early scintigraphic findings [8,21]. Because scintigraphy was performed 5 h after CLP whereas tissue and biochemical analyses were obtained only at the terminal time point, these observations demonstrate concordance across imaging, histological, and molecular domains rather than a temporal or causal sequence. Progressive body-weight loss was likewise compatible with increasing systemic illness burden and the skeletal-muscle wasting described in experimental sepsis [38,39]. However, body weight should be regarded as a nonspecific longitudinal marker rather than a specific indicator of renal injury.

Several limitations should be acknowledged. Although the CLP model reproduces key features of polymicrobial sepsis, it does not fully reflect the biological and clinical heterogeneity of human sepsis. Direct hemodynamic variables, absolute renal blood flow, and renal microvascular perfusion were not measured; therefore, the physiological basis of the reduced R/A ratio remains inferential. The R/A ratio was evaluated in a single experimental cohort and requires external validation. Because biochemical analyses were restricted to animals surviving to the scheduled day-5 terminal sampling, the biomarker findings may have been influenced by survivor-selection bias and may not represent the full spectrum of disease severity in the original cohort. Multivariable Cox regression including biochemical markers was not feasible because these variables were measured only at the terminal experimental time point, which would have introduced temporal and survivor-selection bias. Finally, the exclusive use of female rats represents an additional limitation and restricts the generalizability of the findings across sexes. Although female rats are not inherently more variable than males despite concerns regarding estrous cycle-related variability [40], evidence for sex-related effects in sepsis remains heterogeneous. Some studies have reported sex-dependent physiological and organ-specific responses [41], whereas others found no significant association with selected outcomes [42,43]. Therefore, sex-specific effects should be evaluated in studies including both sexes.

Accordingly, these findings should be regarded as hypothesis-generating rather than evidence of clinical utility. The early R/A ratio should currently be considered an experimental imaging marker associated with disease severity and mortality. Prospective human studies incorporating synchronized imaging and biomarker measurements, comprehensive hemodynamic assessment, prespecified multivariable models, and external validation are needed to determine whether renal perfusion scintigraphy provides clinically meaningful incremental prognostic information.

## Figures and Tables

**Table 1:** Survival status of experimental groups over 5 days.

	Baseline Animal Count (*n*)	Surviving Animals-Day 1	Surviving Animals-Day 2	Surviving Animals-Day 3	Surviving Animals-Day 4	Surviving Animals-Day 5
Control	30	30	30	30	30	**30**
Mild Sepsis	30	22	21	18	18	**17**
Severe Sepsis	30	20	15	12	7	**6**

**Table 2:** Univariable Cox proportional hazards regression analysis for the association between the kidney-to-aorta (R/A) activity ratio and 5-day mortality.

Variable	B	SE	Wald χ^2^	df	*p* Value	Hazard Ratio (HR)	95% CI for HR
Kidney-to-aorta (R/A) activity ratio	−5.384	1.052	26.210	1	<0.001	0.005	0.001–0.036

## Data Availability

The data that support the findings of this study are not publicly available due to ethical reasons, but are available from the corresponding author upon request.

## References

[1] Singer M., Deutschman C.S., Seymour C.W., Shankar-Hari M., Annane D., Bauer M., Bellomo R., Bernard G.R., Chiche J.D., Coopersmith C.M. (2016). The third international consensus definitions for sepsis and septic shock (Sepsis-3). JAMA.

[2] Angus D.C., Van der Poll T. (2013). Severe sepsis and septic shock. N. Engl. J. Med..

[3] Wiersinga W.J., van der Poll T. (2022). Immunopathophysiology of human sepsis. eBioMedicine.

[4] Rudd K.E., Johnson S.C., Agesa K.M., Shackelford K.A., Tsoi D., Kievlan D.R., Colombara D.V., Ikuta K.S., Kissoon N., Finfer S. (2020). Global, regional, and national sepsis incidence and mortality, 1990–2017: Analysis for the Global Burden of Disease Study. Lancet.

[5] World Health Organization (2024). Sepsis [Internet].

[6] Evans L., Rhodes A., Alhazzani W., Antonelli M., Coopersmith C.M., French C., Machado F.R., Mcintyre L., Ostermann M., Prescott H.C. (2021). Surviving sepsis campaign: International guidelines for management of sepsis and septic shock 2021. Crit. Care Med..

[7] Zarbock A., Nadim M.K., Pickkers P., Gomez H., Bell S., Joannidis M., Kashani K., Koyner J.L., Pannu N., Meersch M. (2023). Sepsis-associated acute kidney injury: Consensus report of the 28th Acute Disease Quality Initiative workgroup. Nat. Rev. Nephrol..

[8] Peerapornratana S., Manrique-Caballero C.L., Gómez H., Kellum J.A. (2019). Acute kidney injury from sepsis: Current concepts, epidemiology, pathophysiology, prevention and treatment. Kidney Int..

[9] Gomez H., Ince C., De Backer D., Pickkers P., Payen D., Hotchkiss J., Kellum J.A. (2014). A unified theory of sepsis-induced acute kidney injury: Inflammation, microcirculatory dysfunction, bioenergetics, and the tubular cell adaptation to injury. Shock.

[10] Ince C., Mayeux P.R., Nguyen T., Gomez H., Kellum J.A., Ospina-Tascón G.A., Hernandez G., Murray P., De Backer D. (2016). The endothelium in sepsis. Shock.

[11] John A.K., Norbert L., Peter A., Rashad S.B., Emmanuel A.B., Stuart L.G., Charles A.H., Michael J., Andreas K., Andrew S.L. (2012). Kidney disease: Improving global outcomes (KDIGO) acute kidney injury work group. KDIGO clinical practice guideline for acute kidney injury. Kidney Int. Suppl..

[12] Ostermann M., Legrand M., Meersch M., Srisawat N., Zarbock A., Kellum J.A. (2024). Biomarkers in acute kidney injury. Ann. Intensive Care.

[13] Kounatidis D., Tzivaki I., Daskalopoulou S., Daskou A., Adamou A., Rigatou A., Sdogkos E., Karampela I., Dalamaga M., Vallianou N.G. (2024). Sepsis-associated acute kidney injury: What’s new regarding its diagnostics and therapeutics?. Diagnostics.

[14] Jahaj E., Vassiliou A.G., Pratikaki M., Gallos P., Mastora Z., Dimopoulou I., Orfanos S.E., Orfanos P., Lagiou P., Kotanidou A. (2021). Serum neutrophil gelatinase-associated lipocalin (NGAL) could provide better accuracy than creatinine in predicting acute kidney injury development in critically ill patients. J. Clin. Med..

[15] Han M., Zhang D.H., Zhao L., Liu X.G., Wang Y.X., Qin M.Y. (2023). The impact of instant neutrophil gelatinase-associated lipocalin level on the severity of septic acute kidney injury. Eur. Rev. Med. Pharmacol. Sci..

[16] Taylor A.T., Brandon D.C., de Palma D., Blaufox M.D., Durand E., Erbas B., Grant S.F., Hilson A.J., Morsing A. (2018). SNMMI procedure standard/EANM practice guideline for diuretic renal scintigraphy in adults with suspected upper urinary tract obstruction 1.0. Semin. Nucl. Med..

[17] Rittirsch D., Huber-Lang M.S., Flierl M.A., Ward P.A. (2009). Immunodesign of experimental sepsis by cecal ligation and puncture. Nat. Protoc..

[18] Dejager L., Pinheiro I., Dejonckheere E., Libert C. (2011). Cecal ligation and puncture: The gold standard model for polymicrobial sepsis?. Trends Microbiol..

[19] Dear J.W., Kobayashi H., Jo S.K., Holly M.K., Hu X., Yuen P.S., Brechbiel M.W., Star R.A. (2005). Dendrimer-enhanced MRI as a diagnostic and prognostic biomarker of sepsis-induced acute renal failure in aged mice. Kidney Int..

[20] Holthoff J.H., Wang Z., Patil N.K., Gokden N., Mayeux P.R. (2013). Rolipram improves renal perfusion and function during sepsis in the mouse. J. Pharmacol. Exp. Ther..

[21] Manrique-Caballero C.L., Del Rio-Pertuz G., Gomez H. (2021). Sepsis-associated acute kidney injury. Crit. Care Clin..

[22] Lima A., Van Rooij T., Ergin B., Sorelli M., Ince Y., Specht P.A., Mik E.G., Bocchi L., Kooiman K., De Jong N. (2018). Dynamic contrast-enhanced ultrasound identifies microcirculatory alterations in sepsis-induced acute kidney injury. Crit. Care Med..

[23] Harrois A., Grillot N., Figueiredo S., Duranteau J. (2018). Acute kidney injury is associated with a decrease in cortical renal perfusion during septic shock. Crit. Care.

[24] Watchorn J., Huang D., Bramham K., Hutchings S. (2022). Decreased renal cortical perfusion, independent of changes in renal blood flow and sublingual microcirculatory impairment, is associated with the severity of acute kidney injury in patients with septic shock. Crit. Care.

[25] Vella R., Panci D., Carini F., Malta G., Vieni S., David S., Albano G.D., Puntarello M., Zerbo S., Argo A. (2025). Cytokines in sepsis: A critical review of the literature on systemic inflammation and multiple organ dysfunction. Front. Immunol..

[26] Dolmatova E.V., Wang K., Mandavilli R., Griendling K.K. (2021). The effects of sepsis on endothelium and clinical implications. Cardiovasc. Res..

[27] van der Poll T., Shankar-Hari M., Wiersinga W.J. (2021). The immunology of sepsis. Immunity.

[28] Pace J., Paladugu P., Das B., He J.C., Mallipattu S.K. (2019). Targeting STAT3 signaling in kidney disease. Am. J. Physiol.-Ren. Physiol..

[29] Lee S.H., Kim K.H., Lee S.M., Park S.J., Lee S., Cha R.H., Lee J.W., Kim D.K., Kim Y.S., Ye S.K. (2024). STAT3 blockade ameliorates LPS-induced kidney injury through macrophage-driven inflammation. Cell Commun. Signal..

[30] Lin C.K., Tsai Y.H., Kao K.C., Lin C.M., Zhou S.K., Ho M.C., Huang S.Y., Fang Y.H., Chang C.C., Lee W.C. (2023). Serum vascular endothelial growth factor affects tissue fluid accumulation and is associated with deteriorating tissue perfusion and oxygenation in severe sepsis: A prospective observational study. Eur. J. Med. Res..

[31] Tang A.L., Peng Y., Shen M.J., Liu X.Y., Li S., Xiong M.C., Gao N., Hu T.P., Zhang G.Q. (2022). Prognostic role of elevated VEGF in sepsis: A systematic review and meta-analysis. Front. Physiol..

[32] Ow C.P., Trask-Marino A., Betrie A.H., Evans R.G., May C.N., Lankadeva Y.R. (2021). Targeting oxidative stress in septic acute kidney injury: From theory to practice. J. Clin. Med..

[33] Waikar S.S., Bonventre J.V. (2009). Creatinine kinetics and the definition of acute kidney injury. J. Am. Soc. Nephrol. JASN.

[34] Mårtensson J., Bell M., Xu S., Bottai M., Ravn B., Venge P., Martling C.R. (2013). Association of plasma neutrophil gelatinase-associated lipocalin (NGAL) with sepsis and acute kidney dysfunction. Biomarkers.

[35] Md Ralib A., Mat Nor M.B., Pickering J.W. (2017). Plasma Neutrophil Gelatinase-Associated Lipocalin diagnosed acute kidney injury in patients with systemic inflammatory disease and sepsis. Nephrology.

[36] Chen J.J., Lee T.H., Lee C.C., Chang C.H. (2021). Using lipocalin as a prognostic biomarker in acute kidney injury. Expert Rev. Mol. Diagn..

[37] Klementa V., Petejova N., Zadrazil J., Horak P., Proskova J., Langova K., Klementova O., Kanova M., Martinek A., Sigutova R. (2024). Prediction of acute kidney injury development in critically ill septic patients based on NGAL determination. Physiol. Res..

[38] Leduc-Gaudet J.P., Mayaki D., Reynaud O., Broering F.E., Chaffer T.J., Hussain S.N., Gouspillou G. (2020). Parkin overexpression attenuates sepsis-induced muscle wasting. Cells.

[39] Zanders L., Kny M., Hahn A., Schmidt S., Wundersitz S., Todiras M., Lahmann I., Bandyopadhyay A., Wollersheim T., Kaderali L. (2022). Sepsis induces interleukin 6, gp130/JAK2/STAT3, and muscle wasting. J. Cachexia Sarcopenia Muscle.

[40] Becker J.B., Prendergast B.J., Liang J.W. (2016). Female rats are not more variable than male rats: A meta-analysis of neuroscience studies. Biol. Sex Differ..

[41] Kadkhodaee M., Seifi B., Ranjbaran M., Shams S., Delavari F., Najafi A., Sedaghat Z., Khastar H. (2020). The impact of sex differences on renal protective effects of lipopolysaccharide preconditioning in septic shock. Iran. J. Med. Sci..

[42] Hwang T.L., Chen C.Y. (2012). Gender different response to immunonutrition in liver cirrhosis with sepsis in rats. Nutrients.

[43] Ponce-Alonso M., Fernández-Félix B.M., Halperin A., Rodríguez-Domínguez M., Sánchez-Díaz A.M., Cantón R., Muriel A., Zamora J., Del Campo R. (2021). Propensity-score analysis reveals that sex is not a prognostic factor for mortality in intensive care unit-admitted patients with septic bacteremia. Int. J. Infect. Dis..

